# Supplementary material for: A Census and Categorization Method of Epitranscriptomic Marks
Source: Int J Mol Sci. 2020 Jun 30;21(13):4684. doi: 10.3390/ijms21134684 (PMC7370119; doi:10.3390/ijms21134684)
Supplement: Supplementary file 1 [file FigureS1.pdf › supplementary/FigureS1.pdf]

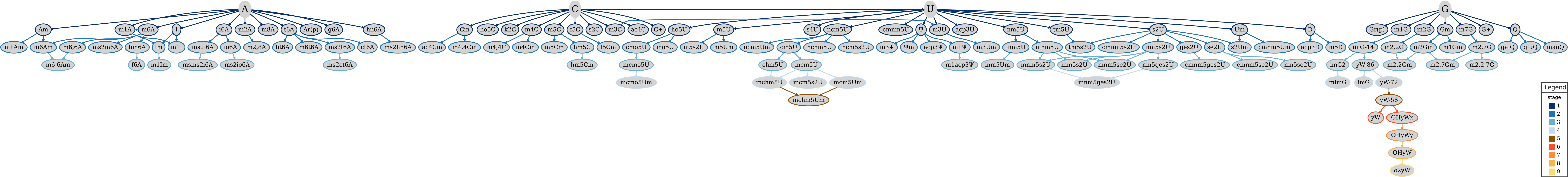
A
Am
m1A
m6A
I
i6A
m2A
m8A
t6A
Ar(p)
g6A
hn6A
m1Am
m6Am
m6,6A
ms2m6A
hm6A
Im
m1I
ms2i6A
io6A
m2,8A
ht6A
m6t6A
ms2t6A
ct6A
ms2hn6A
m6,6Am
f6A
m1Im
msms2i6A
ms2io6A
ms2ct6A
C
Cm
ho5C
k2C
m4C
m5C
f5C
s2C
m3C
ac4C
C+
ac4Cm
m4,4Cm
m4,4C
m4Cm
m5Cm
hm5C
f5Cm
hm5Cm
U
ho5U
m5U
s4U
ncm5U
cmnm5U
Ψ
m3U
acp3U
nm5U
tm5U
s2U
Um
D
cmo5U
mo5U
m5s2U
m5Um
ncm5Um
cm5U
nchm5U
ncm5s2U
m3Ψ
Ψm
acp3Ψ
m1Ψ
m3Um
inm5U
mnm5U
tm5s2U
cmnm5s2U
nm5s2U
ges2U
se2U
s2Um
cmnm5Um
acp3D
m5D
mcmo5U
chm5U
mcm5U
m1acp3Ψ
inm5Um
mnm5s2U
inm5s2U
mnm5se2U
nm5ges2U
cmnm5ges2U
cmnm5se2U
nm5se2U
mcmo5Um
mchm5U
mcm5s2U
mcm5Um
mnm5ges2U
mchm5Um
G
Gr(p)
m1G
m2G
Gm
m7G
G+
Q
imG-14
m2,2G
m2Gm
m1Gm
m2,7G
galQ
gluQ
manQ
imG2
yW-86
m2,2Gm
m2,7Gm
m2,2,7G
mimG
imG
yW-72
yW-58
yW
OHyWx
OHyWy
OHyW
o2yW
Legend
stage
1
2
3
4
5
6
7
8
9
